# ViT-Stain: Vision transformer-driven virtual staining for skin histopathology via global contextual learning

**DOI:** 10.1371/journal.pone.0341311

**Published:** 2026-02-02

**Authors:** Muhammad Altaf Hussain, Muhammad Asim Waris, Muhammad Usman Akram, Muhammad Jawad Khan, Muhammad Zeeshan Asaf, Amber Javaid, Syed Omer Gilani, Fawwaz Hazzazi

**Affiliations:** 1 Department of Biomedical Engineering and Sciences, School of Mechanical and Manufacturing Engineering, National University of Sciences and Technology (NUST), Islamabad, Pakistan; 2 Department of Computer and Software Engineering, College of Electrical and Mechanical Engineering, National University of Sciences and Technology (NUST), Islamabad, Pakistan; 3 Department of Pathology, School of Health Sciences, National University of Sciences and Technology (NUST), Islamabad, Pakistan; 4 Department of Electrical, Computer, and Biomedical Engineering, Abu Dhabi University, Abu Dhabi, United Arab Emirates; 5 Department of Electrical Engineering, College of Engineering, Prince Sattam Bin Abdul Aziz University, Al-Kharj, Saudi Arabia; Institut Sains dan Teknologi Terpadu Surabaya, INDONESIA

## Abstract

Current virtual staining approaches for histopathology slides use convolutional neural networks (CNNs) and generative adversarial networks (GANs). These approaches rely on local receptive fields, struggle to capture global context, and long-range tissue dependencies. This limitation can introduce artifacts in fine textures and cause loss of subtle morphological details. We propose a novel vision transformer-driven virtual staining framework (ViT-Stain) that translates unstained skin tissue images into hematoxylin and eosin (H&E)-equivalent images. The transformer’s self-attention enables ViT-Stain to capture long-range dependencies, preserve global context, and maintain fine textures. We trained ViT-Stain on the E-Staining DermaRepo dataset, which pairs unstained and H&E-stained whole-slide images (WSIs). We validated our model using metrics including SSIM, PSNR, FID, KID, LPIPS, and a novel histology-specific fidelity index (HSFI). Three board-certified pathologists provided feedback for qualitative evaluations. ViT-Stain outperforms leading CNN and GAN models, including Pix2Pix, CycleGAN, CUTGAN, and DCLGAN. It achieves an overall diagnostic concordance of 85% with virtual H&E-stains (Fleiss’ κ = 0.88). However, the model requires longer training (about 93 hours on A100 GPUs) and inference times (about 2.9 minutes). Our work advances AI-driven diagnostic reproducibility for high-fidelity clinical settings and aligns with the World Health Organization (WHO) global health goals.

## Introduction

Histopathological staining with hematoxylin and eosin (H&E) forms the cornerstone of pathology. Preparing H&E slides, however is tedious, time-consuming, and expensive, involving costly reagents [1]. Deep learning (DL) has now made it possible to perform digital virtual staining of unlabeled images, potentially automating this process [2]. Digital virtual staining offers a potential key to a more environmentally sustainable, rapid, and inexpensive alternative to established paradigms. Virtual staining is pivotal in dermatopathology, in which skin diseases [3] represents a large global disease burden, as a major causative factor in non-fatal worldwide morbidity.

Earliest virtual-staining approaches employed image-to-image translation frameworks under computer vision, underpinned by generative adversarial networks (GANs). Isola et al. introduced Pix2Pix, a conditional GAN (c-GAN) that sets a supervised connection between target and source images [4]. Such models, rooted in convolutional neural networks (CNN) learn general mappings but typically require pixel-aligned pairs for training and often produce localized artifacts upon projection over complex tissue structures. This limitation has been mitigated, to some extent, by the cycle-consistent GAN (CycleGAN), which uses unpaired datasets as well as cyclic consistency losses [5] that maintain content continuity across two domains. However, without strong alignment constraints, CycleGAN can easily introduce unrealistic texture or color shifts and often fails to preserve fine-grained details in applications.

Park et al. [6] introduced contrastive unpaired translation GAN (CUTGAN), which is a modification of CycleGAN, replacing the cyclic consistency constraint with a patch-based contrastive loss. CUTGAN optimizes common features between related input and output image parts in a standard way. CUTGAN shows better quality in staining, is more lightweight, and faster compared to CycleGAN [1]. However, CUTGAN and other methods are designed to operate on individual patches and thus lack explicit global context, often resulting in aggravating artifacts or subtle distortions of morphological patterns. Very recently, Asaf et al. [7] proposed dual contrastive learning GAN (DCLGAN). DCLGAN capitalizes on the similarity of real H&E and virtual stains by utilizing dual generators and discriminators. On the E-Staining DermaRepo skin dataset [8], DCLGAN achieved significantly lower Fréchet Inception Distance (FID)/ Kernel Inception Distance (KID), enabling the generation of realistic virtual stains. However, DCLGAN still relies on CNN encoders and decoders with a limited receptive field; therefore, its global spatial coherency is weakly enforced across the entire tissue image.

Vision Transformers (ViTs) define a new paradigm for image modeling, intrinsically using self-attention mechanisms to capture global contextual patterns and long-range dependencies. It was reported that even a pure transformer model [9] can yield remarkable results on image recognition benchmarks by treating an image as a sequence of patches. Every patch in a ViT may attend to every other patch in the image, thus enabling the modeling of long-range structure, which a CNN struggle to preserve. This capability of global modeling is very important for virtual histology. It is possible to empower a ViT-based translator to learn steady staining patterns over extensive tissue areas, preserve texture continuity, as well as guaranteed color correction. In contrast with CNN/GAN models, which emphasize local receptive field as well as partial texture mappings, a ViT implements global contextual patterns, long-range dependencies, as well as structural consistency across the whole image.

### Research contributions

This work presents four main contributions, including **(i)** ViT-Stain, a vision transformer-driven virtual staining framework for skin histology optimization, utilizing the E-Staining DermaRepo dataset [8], **(ii)** a novel histology-specific fidelity index (HSFI) to quantify the diagnostic fidelity of staining models beyond perceptual metrics, **(iii)** clinical validation through pathologist ratings and Turing tests, and **(iv)** diagnostic potential to classify various skin lesions.

During both quantitative and qualitative evaluations, ViT-Stain outperforms Pix2Pix, CycleGAN, CUTGAN, and DCLGAN in artifact reduction, diagnostic fidelity, and preservation of subtle morphological details, but with computational and single-institutional dataset trade-offs. These results indicate that ViT-Stain more realistically replicates H&E morphology than GANs. The global self-attention mechanism in ViT-Stain overcomes the shortcomings of previously used CNNs/GANs, which rely on localized receptive fields and partial texture mappings.

### Organization of the paper

The remainder of this paper is developed in six sections. The related work (section II) highlights tissue staining, GANs, and transformers. Materials and methods (Section III) outline the implementation and training strategies for ViT-Stain/GANs. The experimental results (Section IV) provide detailed quantitative and qualitative evaluations, as well as an analysis of the computational cost and diagnostic potential. The experimental results are adequately discussed in Section V. The limitations and future work have been endorsed in Section VI, followed by the conclusion (Section VII).

## Related work

Initially, virtual staining techniques relied on CNNs trained with paired images. Li et al. [10] used a U-Net based c-GAN (Pix2Pix) to map unlabeled brightfield images of rat artery to H&E-stained versions. Pathologist evaluations revealed virtually no discernible difference, and key morphometric measurements (intima thickness and area) differed by only a few percent. Likewise, Khan et al. [11] systematically compared several Pix2Pix variants on prostate tissues, finding that a dense U-Net encoder achieved a mean structural similarity index measure (SSIM) of ~0.746, compared to 0.725 for a baseline network. These studies illustrate that supervised CNNs can achieve high fidelity when exact input-output registration is available. In such paired settings, Pix2Pix-style networks tend to preserve overall histology details, but they depend critically on perfectly aligned training pairs [12].

Unpaired GANs have also been investigated to overcome the dependence of virtual staining on paired data. Koivukoski et al. [13] applied an unpaired CycleGAN on prostate slides and observed an increased structural realism by adding a paired Pix2Pix step. Salido et al. [14] compared Pix2Pix, CycleGAN, and CUTGAN in multispectral breast images. They reported that CycleGAN achieves the highest SSIM (~0.95) and the lowest color discrepancy between the real and virtually stained images. However, CycleGAN is computationally demanding due to its two-sided mapping and may blur small features. On the contrary, CUTGAN includes one generator with patch-based contrastive loss, handles content better, and converges faster than CycleGAN. Recent works [7] integrate the concept of contrastive learning in a deeper way, such as DCLGAN. The DCLGAN maximizes mutual information between real and virtual H&E patches, yielding much lower FID (~80) and KID (~0.022). De Haan et al. [15] illustrated that the addition of an image-registration sub-network to a Trans-UNet GAN significantly improved staining consistency on autopsy samples compared to vanilla Trans-UNet and CycleGAN. CycleGAN, CUTGAN, and DCLGAN can produce plausible stains, but they often trade off subtle histological details.

Meanwhile, transformer architectures are gaining traction in medical imaging due to modeling of global context. This is beneficial for tasks such as segmentation and synthesis [16]. Chen et al. [17] demonstrated marked dice score improvements in multi-organ segmentation using a hybrid Transformer U-Net. Other works [1] have fused contrastive translation with interpretability. For example, E-CUT adds saliency losses to CUT. However, purely transformer-based staining models remain scarce. For example, ViT-GAN demonstrates general translation with ViT alone, but not yet in histology. This critical gap, as shown in Table 1, highlights the need for hybrid ViT approaches such as our proposed ViT-Stain. ViT-Stain captures global patterns, long-range dependencies, and structural consistency. It also overcomes prior limitations of CNN and GAN through self-attention and a hybrid encoder–decoder, translating unstained skin images into virtual stains.

**Table 1:** Summary – Related work on various virtual staining and image to image translation models.

Model	Year	Architecture	Training Strategy	Dataset Used	Reported Metrics
Pix2Pix [4]	2017	CNN generator (U-Net) + Patch-GAN discriminator	Paired (supervised)	Rat carotid artery, prostate slides	Peak signal to noise ratio (PSNR) ~22–24 dB; SSIM ~0.72–0.75; error <13% in vessel measure
CycleGAN [5]	2017	GAN (two U-Net generators)	Unpaired (unsupervised)	Prostate tissue, breast multispectral, autopsy tissue	SSIM up to ~0.95; qualitative realness; FID ∼(high)
CUTGAN [6]	2020	GAN with single U-Net generator + patch-wise contrastive loss	Unpaired	Breast multispectral, photoacoustic liver	Similar SSIM to CycleGAN
DCLGAN [7]	2024	Dual-generator GAN with contrastive losses	Unpaired	Skin histology	FID ~ 80, KID ~ 0.022 (virtual vs real H&E); high pathologist agreement
Trans-UNet [15]	2024	Hybrid Transformer–CNN encoder/ decoder	Paired (supervised)	Autopsy tissue	Better nuclear morphology preservation than CycleGAN
E-CUT [1]	2024	CUT + Explainability (saliency)	Unpaired	Label-free photoacoustic liver	Expert evaluation: 98% sensitivity vs 94.8% (classification step); visual similarity

## Materials and methods

### Dataset

The E-Staining DermaRepo dataset [8] contains 87 unstained whole-slide images (WSIs) and their H&E-stained counterparts of skin biopsies, all curated under an advarra institutional review board (IRB) protocol. Full patient de-identification was performed in accordance with health insurance portability and accountability act (HIPAA) standards [18]. Notably, 20% of the slides contain multiple tissue sections. This creates 104 unique unstained-to-H&E pairs. Skin biopsy samples are from 15 males and 7 females, aged 34–83 years (median, 67.71 years). All participants provided informed consent. The dataset comprises tissue from both normal skin and various pathological conditions, including basal cell carcinoma (BCC), squamous cell carcinoma (SCC), intra-epidermal carcinoma (IEC), and inflammatory dermatoses. Tissue morphology includes normal samples (n = 47), carcinomas (n = 40), and inflammatory dermatoses (n = 17). All tissue samples were imaged under a Leica Aperio AT2 brightfield microscope at 20 × magnification (0.50 µm/pixel resolution). To minimize inter-slide variability, standardized Kohler illumination was used [19], resulting in high-resolution slides ranging from 0.22 to 1.7 gigapixels. To reduce batch effects, the slides were processed within 6 months using identical H&E staining protocols.

WSIs were divided into 512 × 512-pixel patches by a sliding window approach with an overlap of 256-pixel in both horizontal and vertical directions. This approach guarantees that every area from the original slide will appear multiple times. This results in smoother boundaries when reconstructing WSIs, and maintains a good balance between coverage and redundancy [20]. To avoid data leakage, all image patches from the same slides are assigned to only one split. To ensure diagnostic consistency, a board-certified pathologist concorded unstained and H&E-stained WSIs, rejecting 5% of the slides due to folding or staining artifact issues. Background regions were excluded [21–22] using adaptive thresholding as a quality control step. In particular, the regions where the intensity variance within the patch (intensity ≤ 10%) were excluded. Hence, only those patches containing tissues have been retained for further analysis. After this filtering, the dataset contained 12,450 usable patches (6,225 unstained and H&E pairs). Finally, patches were divided [23] into training (n = 9,960, 80%) and testing (n = 2,490, 20%) sets, representing all diagnostic categories (normal, carcinomas, dermatoses) proportionally.

### Methodology

**Fig 1:**
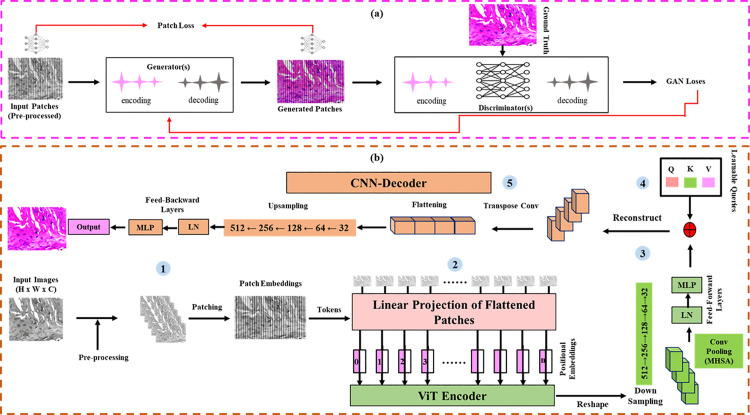
Two classes of staining frameworks shown in a top-to-bottom order. The top image **(a)** displays a classic GAN architecture featuring its generator(s), discriminator(s), and associated losses. The bottom image **(b)**, features the ViT-Stain architecture, having given insight into its encoder-decoder hybrid configuration, along with describing its five core modules responsible for this work.

The proposed methodology consists of three main steps: pre-processing to standardize the data; training staining frameworks, i.e., ViT-Stain and GANs, for effective optimization and convergence as highlighted in Fig 1, and finally, patch inference to obtain the stitched and seamless image.

### Pre-processing

Normalization, registration, and augmentation, a three-phase pre-processing pipeline, standardize the input. This integrated approach normalizes data, enhances generalizability, minimizes potential bias, and also yields an affluent set of training samples.

### Normalization

To reduce inter-sample variation, we globally and per-channel standardize the raw intensities of the images. Specifically, we perform a z-scoring normalization for each RGB channel to ensure a zero mean and a variance of one [24]. This global intensity standardization centers the data and reduces the dynamic-range differences across images. After standardization, we further address color variability by performing stain normalization through histogram matching. In this step, the color histograms of unstained patches are matched to those of a reference H&E-stained template by aligning their intensity distributions [25]. This approach maintains the multimodal nature of the H&E staining more effectively than simple linear scaling or per-channel shifts, and corrects complex stain variations while preserving tissue structures.

### Registration

Standard procedure in tissue examinations sets the uppermost layers, like the epidermis and its sublayers at the top, and the deeper layers, such as the subcutaneous tissue or hypodermis at the bottom. To ensure that the stained and unstained images are correctly aligned, presents a unique challenge due to physical tissue deformation during the staining process. There is a higher possibility of misalignment in space due to difficulties in placing the tissue slides under the microscope at the exact position, or as a result of staining artifacts. Therefore, we carry out spatial registration to align them. We employ the scale invariant feature transformation (SIFT), a feature detection algorithm that is invariant to rotation, depth, illumination, or scale. Thus, SIFT enabled us to establish some key points in both images. We utilize these matching key points to compute the homography matrix. This matrix aligns unstained tissue image with the stained tissue image. The registered images enable us to learn the nonlinear mapping between unstained and stained tissues.

### Augmentation

We apply aggressive on-the-fly augmentation to improve robustness. During training, we randomly rotate each patch (0°, 90°, 180°, or 270°) and flip it horizontally or vertically. These augmentations enlarge the effective training set and promote learning of orientation- and contrast-invariant features. Prior research [26] shows that geometric and photometric transforms reduce overfitting and close the gap between training and test sets. In practice, random rotations, flips, and contrast jitter enhance model generalization to unseen tissue, encouraging invariance to orientation and staining contrast.

### Training of staining frameworks: ViT-Stain & GANs

Each framework was trained on NVIDIA A100 (80GB VRAM) hardware, using equally weighted composite loss functions for pixel, perceptual, adversarial, fidelity, reconstruction, and contrastive terms, with systematically optimized hyperparameters. Hyperparameters were set by grid search over learning rate, β₁/β₂ ratios, and loss weights, followed by manual adjustment to stabilize convergence and maximize staining fidelity.

### ViT-Stain

Various ViTs were studied for our virtual staining task, as depicted in Table 2. However, given the architecture, fidelity requirements, global context capturing, suitability for high-resolution biomedical images, integration and compatibility for a CNN decoder, ViT-base was chosen [9] and its hybrid encoder–decoder architecture tailored for high-fidelity virtual staining of unstained skin histopathology as per the specifications noted in Table 3.

**Table 2:** Transformer and ViTs considered for our virtual staining framework.

Model	Strengths	Limitations
ViT-Base [9]	Simple, proven baseline; 12 heads, 12 layers, 768-dimensional embedding	Requires large datasets; limited inductive bias
Swin Transformer [27]	Hierarchical, shift-window attention; better for dense pixel tasks	Slightly complex to integrate into a hybrid ViT-CNN
Pyramid Vision Transformer [28]	Spatial reduction in attention for high-resolution images; good for segmentation	Slight performance drop on fine textures compared to Swin
Data-efficient Transformer [29]	Light-weight, needs less data; trained with distillation	Marginally less expressive than full ViT-Base or Swin
SegFormer [30]/ UPerFormer [31]	State-of-the-art in medical segmentation; good for dense mapping tasks	More suitable for segmentation than regression-style image synthesis

**Table 3:** Key modifications in ViT-base and its hybrid encoder–decoder architecture tailored for high fidelity virtual staining.

Components	Default ViT-Base	ViT-Stain
Input size	224 × 224	512 × 512
Patch size	16 × 16	16 × 16
Number of patches (tokens)	196 (14 × 14 grid)	1024 (32 × 32 grid)
Decoder	Classification head	Transposed CNN + U-Net skip connections
Positional embedding	Pre-trained or random	Fully learned for domain-specific layout
Loss functions	Cross-entropy (classification)	L_1_ + Perceptual + GAN + HSFI
Application	Image classification	Virtual staining (image translation)

ViT-Stain encoder, as referred from equations (1) to (12), begins by dividing each unstained RGB tissue patch (*512 × 512*) into non-overlapping tokens (*16 × 16*) for the ViT-encoder, resulting in *1,024* total tokens per *512 × 512* patch, i.e., *N = (512/16)²*. Each patch with three color channels is then flattened and projected linearly into a 768-D embedding vector (*d*), forming a token sequence *X ∈ R¹º²⁴*^*×*^*^7^⁶⁸*. Learnable positional embeddings were added to preserve spatial configuration, as in the original ViT. Take an input image *X ∈ R*^*H×W×3*^ *(H = W = 512)*, partition it into non-overlapping patches (*P × P × 3*) with *P = 16*. Denote the *i*-th patch as *p*_*i*_ *∈ R*^*P×P×3*^,


$$x_{i}\;=vec\;(p_{i})\in R^{P^{{}^{2}}\times 3}=R^{768},i=1,\ldots ,1024$$ (1)

$$e_{i}\;=\;\;P_{e}x_{i}\;+\;\;b_{e}\;\in R^{768}$$ (2)

$$E=[e_{1},e_{2},\ldots ,e_{1024}]\in R^{768\times 1024}$$ (3)

$$T^{\left(0\right)}=E+P_{pos}\;\in \;\;R^{768\times 1024}$$ (4)

$$LN(T_{j})=\;\frac{T_{j}-\mu_{j}}{\sqrt{{\sigma_{j}}^{2}+\;\;\in }}\;\gamma +\beta$$ (5)

where *e*_*i*_ are linear patch embeddings, *P*_*e*_ are learnable projections, *E* is stacked to form, *T*^*(0)*^ is the initial token sequence, *P*_*pos*_ are learnable positional embeddings, and LN is the layer normalization having mean (*µ*_*j*_) and variance (*σ*_*i*_).

The resulting 1024-token sequence is fed through 12 transformer encoder blocks. In each block, multi-head self-attention (MHSA) with multiple heads, enabling parallel attention over different feature sub-spaces, computes weighted correlations between all patches. The encoder down-samples the final token sequence into a *32 × 32* feature map (since *1,024 = 32*^*2*^) via non-overlapping *16 × 16* patches (*512 → 256 → 128 → 64 → 32*) and convolves with non-linearities to reconstruct the color image. Formally, for an input *X*, with *N = 1,024*, *d = 768*, queries (*Q*), keys (*K*), and values (*V*), the attention output head is concatenated and projected. Each attention block is followed by a two-layer multi-layer perceptron (MLP) with a hidden size of *3,072*, Gaussian error linear unit (GELU) activation in each sub-layer, and residual layer-normalization (LN) as in [9].

The ViT-Stain encoder employs this global MHSA mechanism to directly assess pairwise interactions among all patch tokens within an input context window. This approach allows it to model long-range dependencies across multiple tissue regions as opposed to the local operation in CNN kernels [32–33]. A single MHSA layer computes a weighted sum of values (V) for every token, where attention weights to calculate such values depend on all tokens. This forms global connectivity within a single step. This is opposed to a traditional CNN, which has a typical expansion in the size of the receptive field as the network deepens. Long-range dependencies are indirectly resolved by multiple convolutions in a local manner. This is important for three central reasons in virtual staining. First, histological samples present morphologically informative structures that come at different scales, such as epidermal and dermal boundaries, glandular architectures, and tumor margins. The structure’s look at one place informs the expected staining appearance at a distant location. Global attention allows for direct conditioning of context such that a token can incorporate evidence from other tokens regardless of spatial distance. Global attention helps attain long-range structure coherence that agrees with tissue-level architecture in a single pass. Second, many of the local textures are inherently ambiguous; for instance, eosinophilic cytoplasm versus extracellular matrix. This means that neighboring tissues together with layer depth, are required to clear the difference between them. The attention weights introduce an influence on the model as it pays more attention to relevant distant tokens when reconstructing local textures; hence, the inconsistencies and hallucinations are resolved. Finally, MHSA has content-adaptive mechanisms. An input content informs the attention weights to compile different sets of contexts for a token in different cases, as in tumors versus benign cases. This adaptability improves generalization across diverse tissue patterns. In contrast, fixed convolutional filters assess the same fixed neighborhood.

Let a token sequence *T*^*(i-1)*^ *∈ R*^*D×N*^ be the input to the *i – th* block. Denote H = 12 heads, each of dimension $d_{h}=\frac{D}{H}=64$. For head h:


$$Q^{\left(i,h\right)}={P_{q}}^{\left(i,h\right)}T^{\left(i-1\right)},\;\;\;K^{\left(i,h\right)}={P_{k}}^{\left(i,h\right)}T^{\left(i-1\right)},\;\;\;V^{\left(i,h\right)}={P_{v}}^{\left(i,h\right)}T^{\left(i-1\right)}$$ (6)

where $T^{\left(i-1\right)}=LN(T^{\left(i-1\right)})\in R^{D\times N}$ and ${P_{q}}^{\left(i,h\right)},\;\;{P_{k}}^{\left(i,h\right)},\;\;{P_{v}}^{\left(i,h\right)}\;\in R^{\;d_{h}\;\times \;D}$


$$A^{\left(i,h\right)}=Softmax\;(\frac{(Q^{\left(i,h\right)})\;{\left(K^{\left(i,h\right)}\right)}^{T}}{\sqrt{d_{h}}})\;V\in R^{N\times N}$$ (7)

where $O^{\left(i,h\right)}=V^{\left(i,h\right)}{A^{\left(i,h\right)}}^{T}\in R^{d_{h}\times N}$.

Concatenate all the heads and project them back to dimension *D*:


$$O^{\left(i\right)}=Concat(O^{\left(i,1\right)},\ldots ,O^{\left(i,H\right)})\in R^{D\times N},\;MHSA^{\left(i\right)}(T^{\left(i-1\right)})={P_{o}}^{\left(i\right)}O^{\left(i\right)}$$ (8)

where ${P_{o}}^{\left(i\right)}\;\in R^{D\times D}$.


$$T{}^{\prime \left(i\right)}=T^{\left(i-1\right)}+MHSA^{\left(i\right)}(LN(T^{\left(i-1\right)}))$$ (9)

After a second *LN* on $T\prime^{\left(i\right)}$, apply a two-layer MLP with hidden size *D*_*mlp*_ *= 3,072*:


$$T{}^{\prime \left(i\right)}=LN(T{}^{\prime \left(i\right)})$$ (10)

$$MLP^{\left(i\right)}(T{}^{\prime \left(i\right)})={P_{2}}^{\left(i\right)}\sigma ({P_{1}}^{\left(i\right)}T{}^{\prime^{\left(i\right)}}+{b_{1}}^{\left(i\right)})+{b_{2}}^{\left(i\right)}$$ (11)

where ${P_{1}}^{\left(i\right)}\;\in \;\;R^{D_{mlp}\times D},{P_{2}}^{\left(i\right)}\;\in \;\;R^{D\times D_{mlp}},{b_{1}}^{\left(i\right)}\;\in \;\;R^{D_{mlp}},{b_{2}}^{\left(i\right)}\;\in \;\;R^{D}.$


$$T^{\left(i\right)}=T{}^{\prime \left(i\right)}+MLP^{\left(i\right)}(LN(T{}^{\prime \left(i\right)}))$$ (12)

We repeated steps from equations (6) to (12) till the last encoder block. The output of the last block is denoted as *T* ^*(12)*^ *∈ R*^*768 × 1024*^.

After MHSA, the 1024-token output (*32 × 32* grid) is reshaped into a spatial feature map of size *32 × 32 × 768*, forming the input to the decoder. The decoder is a CNN that upsamples the encoded features back to *512 × 512* resolution via 4-stage (*32 → 64 → 128 → 256 → 512*) transpose convolution or pixel shuffle. Inspired by U-Net [34], the decoder uses multi-scale feature channels and skip connections to preserve fine details, ensuring that the output image faithfully recovers spatial resolution. Prior to decoding, the network generates 786,432 (*1024 × 768*) intermediate logits, thereby maintaining the full resolution of histological texture.

ViT-Stain’s training strategy employs the AdamW optimizer [35], which includes learnable affine parameters in normalization (*β₁*, *β₂*) and a learning rate, utilizing a cosine annealing schedule [36]. The learning rate was searched in (*1 × 10*^*−5*^, *5 × 10*^*−5*^, *1 × 10*^*−4*^, *5 × 10*^*−4*^), and Adam optimizer parameters were varied with *β1 ∈ (0.5,0.9) and β2 ∈ (0.999,0.9995)*. The best configuration (*3 × 10*^*−4*^, β*1 = 0.9,* β*2 = 0.999*) was then manually refined by early stopping based on validation SSIM. The learning rate is decayed smoothly to zero over 200 epochs via *ηₜ = η*_*0*_ *× 0.5 × (1 + cos (π × t/ T))* where *ηₜ, η*_*0*_, *T,* and *t* are step learning rate (η*ₜ*), initial learning rate (*η*_*0*_), total (*T*), and current (*t*) number of training epochs respectively. This warm-restart schedule helps the model escape sharp minima. All transformer parameters are initialized as in [9], and the decoder’s CNN weights are initialized with Kaiming normal initialization. LN and dropout (with a probability of 0.1) are used in transformer blocks for regularization. We train with a batch size of 8 (across NVIDIA A100 GPU) using mixed-precision arithmetic for speed.

During training, we alternate gradient updates: one step for the discriminator (maximizing its classification loss) and one step for the generator (minimizing the combined loss). The training process was monitored on a held-out validation set to prevent overfitting. We observed that the network reliably learned realistic staining patterns within ~190 epochs. The details of training parameters are provided in Table 4. We trained the network with a composite loss function (*L*). The total loss, as shown in equation (13), is a weighted sum of reconstruction, perceptual quality, adversarial realism, and HSFI.

**Table 4:** The details of precise training parameters, hyperparameters and hardware, etc. used for staining frameworks during training and inference.

Serial	Training Parameters	Precise Settings
1	Number of training patches	9,960
2	Number of testing patches	2,490
3	Size of each training patch	512 × 512 with a 256-pixel overlap between consecutive patches
4	Overlapping between consecutive patches	Yes
5	Token size (ViT-Stain)	16 × 16 non-overlapping patches for the ViT-encoder, resulting into 1,024 token
6	Optimization strategy	Mixed precision arithmetic including hyper-parameter tuning, i.e., AdamW optimizer with cosine annealing (ViT-Stain) and FP16 Mixed Precision (GANs)
7	Learning rate	*3 × 10*^ *− 4*^*, β*_*1*_ *= 0.9* (ViT-Stain) & 0.5 (GANs)*, β*_*2*_ *= 0.999*, decayed smoothly to zero over 200 epochs via cosine annealing.
8	Dropout probability	0.1 during training and Kaiming weight initialization
9	Loss weights (ViT-Stain)	*λ*_*L1*_ *= 1, λ*_*perc*_ *= 0.1, λ*_*GAN*_ *= 0.01 and λ*_*HSFI*_ *= 1*
10	Loss weights (GANs)	*λ*_*L1*_ *= 100, λ*_*patchNCE*_ *= 1 (CUT) & 2 (DCLGAN), λ*_*GAN*_ *= 1, λ*_*identity*_ *= 1 and λ*_*cycle*_ *= 10*
11	Batch size	8
12	Hardware	High-End NVIDIA A100 GPU (80GB VRAM)
13	Number of epoch	200


$$L=\lambda_{L_{1}}L_{1}+\lambda_{perc}L_{perc}+\lambda_{GAN}L_{GAN}+\lambda_{HSFI}L_{HSFI}$$ (13)

*L*_*1*_ (reconstruction) loss measures absolute pixel difference between the synthetic and real stain images. *L*_*1*_ is comparatively less outlier-prone [37] than *L*_*2*_, i.e., mean squared error (MSE). *L*_*perC*_ employs the visual geometry group 19 (VGG 19) network, pre-trained on ImageNet [38], to compute feature reconstruction differences. *L*_*GAN*_ employs a patch-GAN discriminator (*70 × 70*) to distinguish pixel patches as real or fake [39]. The novel *L*_*HSFI*_ introduces a domain-tailored loss function to preserve histological structures (e.g., nucleus/cytoplasm contrast and stain colors). Intuitively, HSFI penalizes mismatches in color distributions and key tissue patterns that are critical for diagnosis. The weights *λ* are tuned so that no single loss dominates in our experiments.

### GAN architectures

Pix2Pix learns one-sided mapping using paired data. It employs one generator (*G*_*P*_) and one discriminator (*D*_*P*_) each. Its *D*_*P*_ is a patch-GAN that classifies image patches for high-frequency structure by employing a c-GAN and *L*_*1*_ (reconstruction) losses as referred from equations (14) to (16), respectively.


$$L_{P}=L_{c-GAN}+\lambda_{L1}L_{1}$$ (14)

$$L_{c-GAN}(G_{P},D_{P})=E\;\;\left[logD_{P}\right]+E\left[log\left(1-D_{P}\;\left(G_{P}\right)\right)\right]$$ (15)

$$L_{1}(G_{P})=E_{\;1,2}[\;∥2-G_{P}\left(1\right)∥{\;}_{1}]$$ (16)

where *λ*_*L1*_ balances adversarial realism against pixel-level accuracy.

CycleGAN extends the GANs to unpaired image-to-image translation by learning a two-sided mapping. It employs two generators (*G*_*Cy1*_*, G*_*Cy2*_) and two discriminators (*D*_*Cy1*_*, D*_*Cy2*_) each. Its dual adversarial approach is augmented with cycle consistency loss and adversarial losses, as shown in equations (17) to (20), respectively.


$$L_{Cy}=L_{GAN}{}^{1,2}+{L_{GAN}}^{2,1}+\lambda_{Cy}L_{Cy}\left({G_{Cy}}^{1,2}\right)$$ (17)

$$L_{GAN}{}^{1,2}=E_{2}\sim p2\;\left[logD_{Cy_{2}}\left(2\right)\right]+E_{1}\sim p1\;\left[log\left(1-D_{Cy_{2}}\left(G_{Cy_{1}}\right)\right)\right]$$ (18)

$${L_{GAN}}^{2,1}=E_{1}\sim p1\;\left[logD_{Cy_{1}}\left(1\right)\right]+E_{2}\sim p2\;\left[log\left(1-D_{Cy_{1}}\left(G_{Cy_{2}}\right)\right)\right]$$ (19)

$$L_{Cy}\left({G_{Cy}}^{1,2}\right)=E_{1}\sim 1\;[∥D_{Cy_{2}}\left(G_{Cy_{1}}\right)-1{∥}_{\;\;1}]+E_{2}\sim 2\;[∥D_{Cy_{1}}\left(G_{Cy_{2}}\right)-2{∥}_{\;\;1}]$$ (20)

where *λ*_*cycle*_ penalizes deviations in forward and backward translations.

CUTGAN is a contrastive learning architecture for unpaired translations and learns one-sided mappings. It employs one generator (*G*_*C*_) and one discriminator (*D*_*C*_) each. CUTGAN focuses on maintaining fine-grained local details by employing a standard adversarial loss and a patch noise contrastive estimation (NCE) loss, as shown in equations (21) to (23), respectively.


$$L_{C}=L_{GAN}+\lambda_{patchNCE}L_{patchNCE}$$ (21)

$$L_{GAN}=E\;\;\left[\log D_{C}\right]+E\;\;\left[log\left(1-D_{C}\left(G_{C}\right)\right)\right]$$ (22)

$$L_{patchNCE}=\frac{\sum_{i}Log\;e^{{\left(\phi \;.\;\;\phi_{\;G_{c}}\right)}_{i}}/\tau }{\sum_{j}Log\;e^{{\left(\phi \;.\phi {\;}_{G_{c}}\right)}_{j}}/\tau }$$ (23)

where *λ*_*patchNCE*_ maximizes mutual information between corresponding unstained and H&E patches, *τ* is a temperature parameter that scales the logits, and the dot product measures similarity in the feature space (*ϕ*).

DCLGAN employs contrastive learning by establishing two-sided mappings, rather than relying on cycle consistency. DCLGAN has four key modules: two generators (*G*_*1*_*, G*_*2*_), two discriminators (*D*_*1*_*, D*_*2*_), two multi-layered perceptrons (*H*_*1*_*, H*_*2*_) having two layers each, and three distinct loss functions, i.e., adversarial, patch NCE, and identity losses that direct model training from (24) to (28).


$$L_{DCL}=\lambda_{GAN}L_{GAN}+\lambda_{patchNCE}L_{patchNCE}+\lambda_{identity}L_{identity}$$ (24)

$${L_{GAN}}^{1,2}=E_{2}\sim p2\left[logD_{2}\left(2\right)\right]+E_{1}\sim p1\;\left[log\left(1-D_{2}\left(G_{1}\right)\right)\right]$$ (25)

$${L_{GAN}}^{2,1}=E_{1}\sim p1\;\left[logD_{1}\left(1\right)\right]+E_{2}\sim p2\;\left[log\left(1-D_{1}\left(G_{2}\right)\right)\right]$$ (26)

$$L_{patchNCE}=\frac{\sum_{i}Log\;e^{{\left(\phi \;.\;\;\phi_{\;G_{DCL}}\right)}_{i}}/\tau }{\sum_{j}Log\;e^{{\left(\phi \;.\phi {\;}_{G_{DCL}}\right)}_{j}}/\tau }$$ (27)

$$L_{identity}\left(G^{1,2}\right)=E_{1}\sim 1\;[∥G\left(1\right)-1{∥}_{\;\;1}]+E_{2}\sim 2\;[∥G_{1}\left(2\right)-2∥{\;}_{1}]$$ (28)

**Fig 2:**
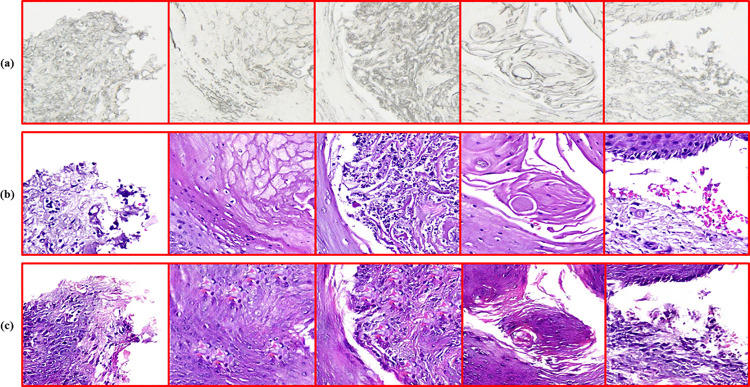
The top row (a) shows the unstained source image patches, while the middle row (b) shows the paired H&E-stained patches, and the bottom row (c) shows corresponding virtual H&E-equivalent image patches generated by the respective staining frameworks.

**Fig 3:**
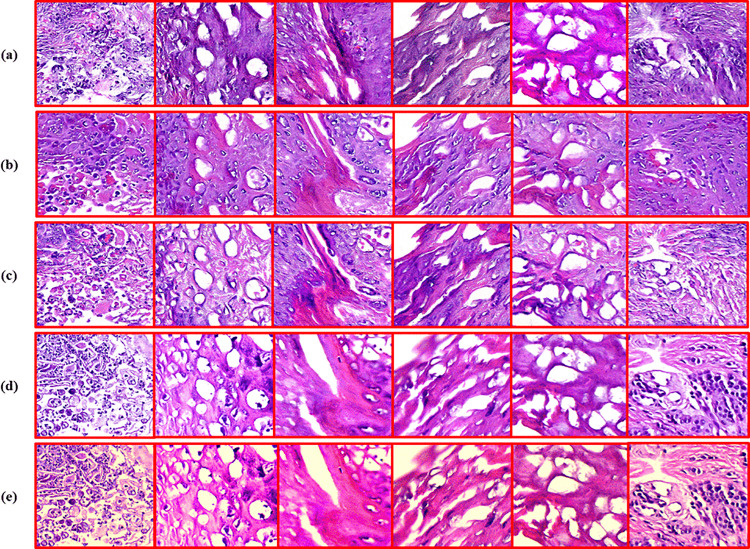
Virtually stained H&E-equivalent image patches generated by the respective staining frameworks are represented in five rows from top to bottom. The top row **(a)** shows the virtually stained patches generated by Pix2Pix, demonstrating low-frequency details. 2^**nd**^ row **(b)** depicts CUTGAN patches, demonstrating weak distributional details. The 3^**rd**^ row **(c)** depicts DCLGAN output patches with slightly lower artifacts and hallucinations than CUTGAN. In 4th row **(d)**, CycleGAN is seen to have good content and stain preservation, while the bottom row **(e)** shows ViT-Stain-generated patches with strong structural coherence, high distributional details, lowest hallucinations, and superior stain specificity.

Each GAN [4–7] optimized with the floating point 16 (FP16) mixed precision [40], learning rate of *3 × 10*^*−4*^*, β*_*1*_ *= 0.5, β*_*2*_ *= 0.999*, and batch size of 8 for 200 epochs. Hyperparameters were determined via a systematic grid search over learning rate, *β*_*1*_*/* β_2_ ratios, and loss-weight combinations as depicted in Table 4, followed by targeted manual refinement to stabilize convergence and maximize staining fidelity. The composite loss functions, in combination with robust optimization techniques, facilitated effective learning of the intricate mappings needed for virtual staining. More importantly, the incorporation of the contrastive loss terms into both CUTGAN and DCLGAN, inspired by recent progress [41], has proved essential for the preservation of context and histological details shown in Figs 2 and 3, respectively.

### Patch inference

**Fig 4:**
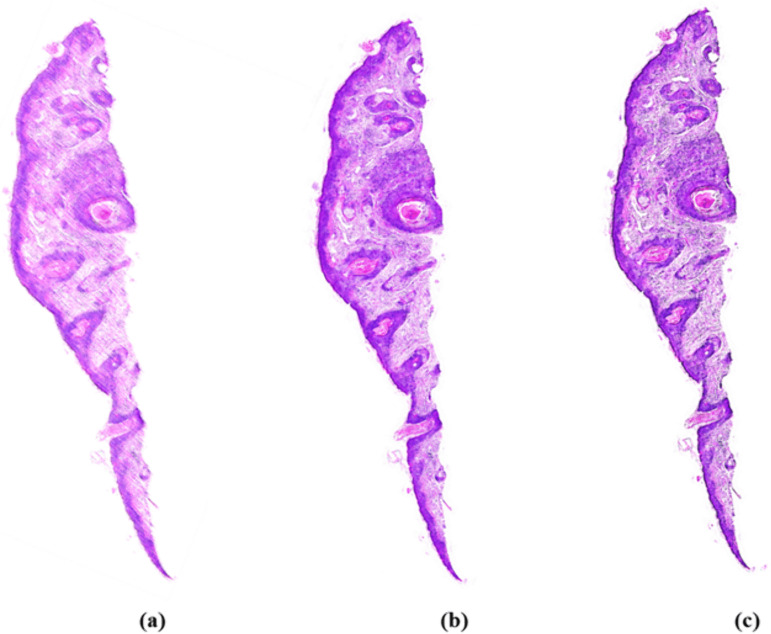
In the left image (a) the virtual staining output is noisy and inconsistent, with visible artifacts. In contrast, the middle **(b)** shows a GAN image and the right **(c)** shows an ADVS image after merging and blending patches. These images are smooth and seamless with negligible edge artifacts.

In inference, virtually generated patches by the respective staining frameworks are merged to reconstruct a complete tissue image. This merger might result in boundary artifacts, a significant issue during inference of neighboring virtual patches as a result of color and contrast variations. Such issues occur during reconstructing WSIs from related patches and might affect the diagnostic integrity of virtual stains. For efficient reduction of such reconstruction issues, we utilize a 50% patch overlapping between two neighboring patches, followed by alpha blending of overlapping areas to allow for smooth, and seamless transfer of pixel intensities [42]. The resultant image, therefore exhibits a smooth transfer at patch boundaries and retains the diagnostic integrity with no textural incoherence and boundary artifacts, as depicted in Fig 4.

## Results & analysis

### Quantitative evaluations

**Fig 5:**
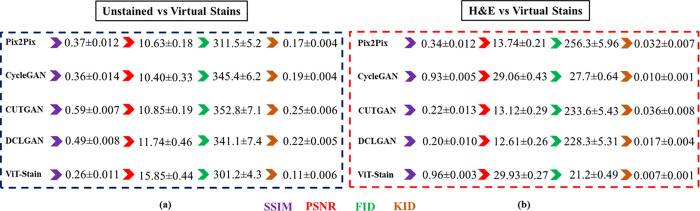
Quantitative results for perceptual and distributional metrics across two image distributions are shown from left to right. The left image **(a)** displays quantitative results between unstained vs virtually generated patches, while the right image **(b)** highlights the same comparison between H&E vs virtually generated patches using the respective staining frameworks.

**Fig 6:**
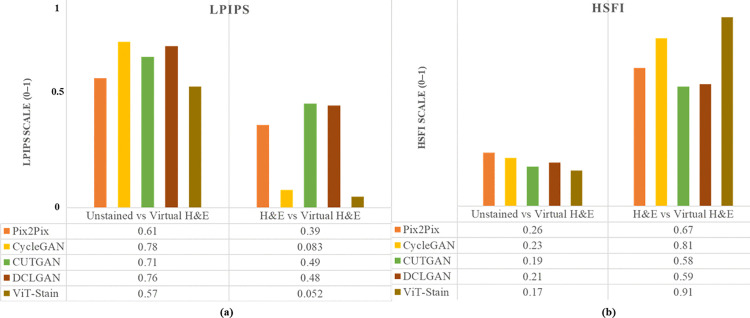
Quantitative results for perceptual error and diagnostic fidelity across two image distributions, unstained vs virtually generated patches, H&E vs virtually generated patches, are shown as bar plots from left to right. The plots on the left **(a)** represent the perceptual error represented by LPIPS, while the right one **(b)** represents diagnostic fidelity (HSFI) of respective staining frameworks.

To evaluate the performance of each virtual staining framework, we used several quantitative measures, including SSIM, PSNR, FID, KID, LPIPS, and HSFI, as well as computational cost and diagnostic classification. The results for SSIM, PSNR, FID, and KID are given in Fig 5. The LPIPS and HSFI obtained from the respective staining frameworks are depicted in Fig 6. Moreover, all reported values are given as mean ± standard deviation with 95% CI, and the p-value ≤ 0.05 was computed using a paired t-test with n = 100 paired patches.

### SSIM & PSNR

SSIM measures image similarities based on changes in structural details, luminance, and contrast. Consistently lower mean SSIM values of 0.26–0.59 across the virtual staining frameworks between unstained and virtually stained images reassured the inherent domain differences and underpinned the necessity for translation of images. When comparing virtual to real H&E images, ViT-Stain achieved a mean SSIM of 0.96 ± 0.003, outpacing Pix2Pix (0.34 ± 0.012), CycleGAN (0.93 ± 0.005), CUTGAN (0.22 ± 0.013), and DCLGAN (0.20 ± 0.010). PSNR quantifies image similarities at the pixel level by comparing exact color and intensity. In terms of PSNR, all virtual staining frameworks generate lower mean PSNR values between unstained and virtually stained images (10.40–15.85 dB), which indicates a domain gap and confirms that the applications of virtual staining frameworks serve as a bridge between the unstained and stained images. Comparing the virtual images to real H&E images, ViT-Stain had a mean score of 29.93 ± 0.27 dB, slightly superior to the CycleGAN with 29.06 ± 0.43 dB, while outperforming those for Pix2Pix, 13.74 ± 0.21 dB, CUTGAN, 13.12 ± 0.29 dB, and DCLGAN, 12.61 ± 0.26 dB, by a significant margin. Collectively, these gains are significant; for example, a 30 dB PSNR gain represents roughly an order of magnitude reduction in mean squared error (*L*_*2*_) and much sharper reconstructions.

### FID & KID

The FID is a measure of distributional similarity and coherence of high-level features, so it is sensitive to textural and structural fidelity. An FID ≤ 25 is equated with perceived realism in computational histopathology. On the other hand, higher mean FID values of 301.2–352.8 between unstained and virtually stained images are indicative of the domain shift between these two kinds of histologies. ViT-Stain also improves in feature-space distribution (21.2 ± 0.49), slightly better than CycleGAN (27.7 ± 0.64). Its mean FID is also way lower as compared to the ranges of 256.3 ± 5.96, 233.6 ± 5.43, and 228.3 ± 5.31 for Pix2Pix, CUTGAN, and DCLGAN, respectively, in virtual versus real H&E. Similarly, KID measures the distributional dissimilarity between images by using the maximum mean discrepancy (MMD) of features from a pre-trained inception-v3 network. The lower value of KID implies that the sets of images are more similar, and vice versa. The higher mean KID values of 0.11–0.25 of unstained and virtually stained images indicate an inherent domain gap, which underlines the requirement of sophisticated translation frameworks. In contrast, the mean KID for ViT-Stain is 0.007 ± 0.001, which is lower than the ranges of 0.032 ± 0.007, 0.010 ± 0.001, 0.036 ± 0.008, and 0.017 ± 0.004 for Pix2Pix, CycleGAN, CUTGAN, and DCLGAN when comparing virtual and real H&E. These close to zero mean KID values of ViT-Stain indicate that its outputs are close to distributional similarities of real H&E. Across both FID and KID, ViT-Stain generates better distributional fidelity, compared to GAN-based methods.

### LPIPS

LPIPS is an evaluation metric that estimates the similarity between two images by comparing deep feature representations. LPIPS mainly focuses on semantic and textural differences. The perceptual realism increases for lower LPIPS values. On the contrary, higher mean LPIPS values for a range of 0.57–0.78 between unstained and virtually stained images highlight the perceptual gap and hint at the usage of strong translation models. However, the LPIPS perceptual error decreased in the virtual versus real H&E case. ViT-Stain achieved an LPIPS score of (0.052 ± 0.001), followed closely by CycleGAN (0.083 ± 0.002). However, the LPIPS scores for Pix2Pix (0.39 ± 0.01), CUTGAN (0.49 ± 0.01), and DCLGAN (0.48 ± 0.01) remained higher when comparing virtual and real H&E. ViT-Stain preserved low-level texture and color much better than GAN outputs, especially Pix2Pix, CUTGAN, and DCLGAN.

### HSFI

This novel index represents how the pathologists diagnose by integrating domain knowledge into clinical scores [43–44]. HSFI integrates weighted assessments of key histological features. This includes nuclear morphology (shape, size, and chromatin texture), and tissue architecture (epidermal layers and stromal organization). It also considers stain consistency by computing mean channel-wise variance across H&E spectral components through color deconvolution. HSFI ranges from 0 to 1, where values above 0 reflect higher diagnostic accuracy. The HSFI in equation (29) gives a holistic measure that closely follows real-world interpretation criteria in histopathology.


HSFI=α(NMS)+β(TAS)+γ(SCS) (29)

where *α, β,* and *γ* represent the optimized weighting coefficients, which are determined by grid search (*0.2, 0.3, 0.4, 0.5*) to achieve the highest agreements with the expert pathologists’ ratings. After cross-validation, the final weights were set to *α = 0.3, β = 0.4, and γ = 0.3*. Meanwhile, NMS, TAS, and SCS stand for the respective scores of nuclear morphology, tissue architecture, and stain consistency.

The lower mean HSFI scores of 0.17–0.26 measured between unstained and virtually stained images indicate the necessity for accurate translation frameworks. On the other hand, the strong performance by ViT-Stain can also be seen in determining diagnostic accuracy when comparing virtual to real H&E images, a critical factor in diagnostic fidelity. Its HSFI (0.91 ± 0.02) stayed significantly higher than Pix2Pix (0.67 ± 0.02), CUTGAN (0.58 ± 0.01), and DCLGAN (0.59 ± 0.01), while CycleGAN was closest at (0.81 ± 0.02).

### Computational cost

**Fig 7:**
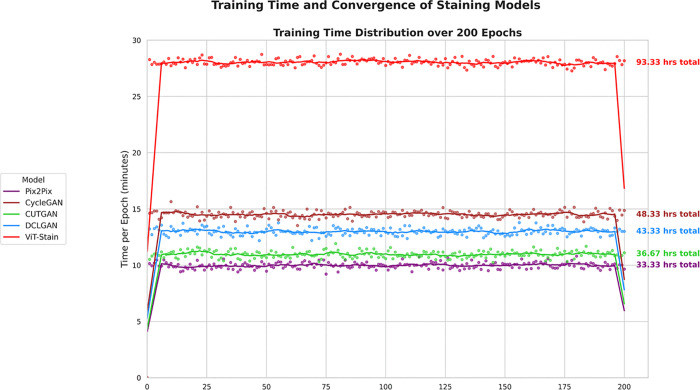
Training time and convergence plots of the respective staining frameworks distributed over 200 epochs. The ViT-Stain plot indicates an initial, continuous, and sharp increase in per-epoch time, but it stabilizes quickly and then follows a sharp decline in per-epoch time at convergence, within ~190 epochs. In contrast, GANs exhibit a moderate initial increase in per-epoch time but demonstrate similar behavior to ViT-Stain during stabilization and convergence, within ~190 epochs.

**Fig 8:**
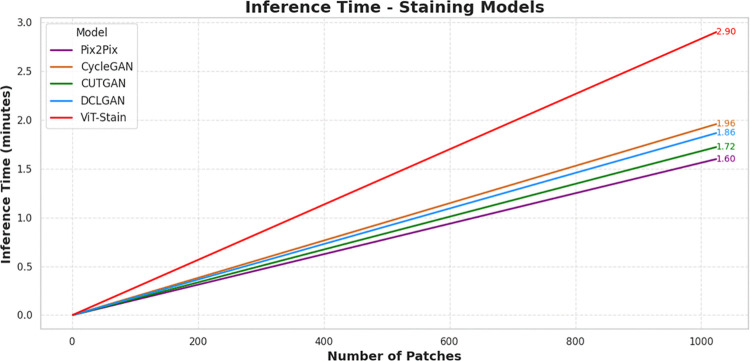
Inference time (latency) behavior of the respective staining frameworks during patch merger. ViT-Stain exhibits higher inference time (latency). In contrast, the behavior of GANs remains largely unchanged, with only slightly different inference times (latency).

Each architecture was trained with identical training conditions, including hardware, setup, batch size, optimization strategy, and number of epochs. As seen in Fig 7, the training times are different among frameworks in the following order: Pix2Pix (~33.33 hours/1.39 days), CUTGAN (~36.67 hours/1.53 days), DCLGAN (~43.33 hours/1.80 days), CycleGAN (~48.33 hours/2.01 days), and ViT-Stain (~93.33 hours/3.89 days). Pix2Pix and CUTGAN are cost-effective models to train because of their simpler architecture and/or optimized loss functions. For DCLGAN and CycleGAN, dual contrastive loss weights, double GAN dynamics, and cyclic consistency add computational overhead for both training and inference times. In contrast, the higher cost of ViT-Stain is due to its complex architecture, computational scale, and token-based processing. Fig 8 highlights that all GANs require ~1.60–1.96 minutes for inference, while ViT-Stain requires ~2.90 minutes.

### Qualitative evaluations

Three board-certified dermatopathologists with a combined 35 years of experience assessed both virtual and real H&E images. The experts were unaware of the image source (whether real H&E or virtual stain), patient identifiers, or any case metadata. The images were presented in pairs. Fifty full WSIs and fifty cropped patches at 20 × magnification were shown to each of the dermatopathologists. Experts used a standardized rubric [45] to rate staining specificity on a 5-point Likert scale. Diagnostic trustworthiness was rated on a binary scale (Yes/No), and artifact detection was graded on a severity scale. To set a baseline for clinical agreement, the same dermatopathologists evaluated the real H&E slides and patches under the same blinded conditions. We calculated pairwise Fleiss’ κ for inter-rater agreement. Diagnostic concordance [46] for each image type is the percentage of cases where a dermatopathologist’s diagnosis matches the reference diagnosis, defined as the majority vote of the H&E consensus panel. For each metric, we report mean ± standard deviation with a 95% CI and a p-value ≤ 0.05. The mean values of the qualitative evaluations by the dermatopathologists are shown in Table 5.

**Table 5:** Qualitative evaluations by board-certified dermatopathologists comparing real and virtual H&E images and patches from each staining framework.

Metric	H&E	Pix2Pix	CycleGAN	CUTGAN	DCLGAN	ViT-Stain
**Stain specificity (5-point Likert Scale)**						
H&E Consistency	4.8 ± 0.1	4.2 ± 0.1	4.4 ± 0.2	3.7 ± 0.5	4.1 ± 0.3	4.6 ± 0.1
Melanin Differentiation	4.7 ± 0.1	3.9 ± 0.5	4.2 ± 0.3	3.3 ± 0.6	3.9 ± 0.4	4.4 ± 0.3
**Diagnostic trustworthiness (Yes/ No %)**						
Nuclear Atypia (Sufficiency)	90 ± 4	70 ± 10	75 ± 5	40 ± 18	50 ± 15	80 ± 5
Tissue Architecture (Sufficiency)	95 ± 2	80 ± 4	90 ± 3	40 ± 8	70 ± 5	90 ± 3
Mitotic Figure Accuracy	85 ± 5	50 ± 10	60 ± 8	30 ± 12	30 ± 15	75 ± 7
**Artifact severity (%)**						
Blurring (None/ Mild/ Moderate/ Severe)	95/ 2/ 2/ 1	60/ 30/ 8/ 2	85/ 10/ 3/ 2	50/ 15/ 10/ 25	60/ 15/ 20/ 5	90/ 7/ 2/ 1
Over-Staining	3 ± 1	15 ± 3	10 ± 2	20 ± 5	20 ± 5	5 ± 1
Hallucinations	5 ± 1	15 ± 3	10 ± 2	45 ± 15	30 ± 10	10 ± 1
**Inter-rater agreement (Fleiss’ κ)**	0.94 ± 0.05	0.80 ± 0.10	0.85 ± 0.08	0.58 ± 0.15	0.68 ± 0.14	0.88 ± 0.08
**Turing test success (%)**	**92** ** ±** ** 2**	**72** ** ±** ** 4**	**81** ** ±** ** 3**	**43** ** ±** ** 7**	**57** ** ±** ** 6**	**85** ** ±** ** 2**

Pix2Pix preserved nuclear atypia (70 ± 10%) and tissue architecture (80 ± 4%) but struggled with artifacts, i.e., blurring (40%), over-staining (15 ± 3%), and hallucinations (15 ± 3%). Pix2Pix achieved good H&E consistency (4.2 ± 0.1) but struggled with melanin differentiation (3.9 ± 0.5). Its Turing test success rate (72 ± 4%) is validated by κ = 0.80 ± 0.10, achieving good inter-rater agreement. CycleGAN achieved good H&E consistency (4.4 ± 0.2) and melanin differentiation (4.2 ± 0.3), both critical features for melanoma diagnosis. It has demonstrated strong diagnostic trustworthiness for nuclear atypia by 75 ± 5% and tissue architecture by 90 ± 3%, but struggled to be accurate in depicting mitotic figures, 60 ± 8%. The minimal blurring, overstaining, and hallucinations were each 10 ± 2%, falling well within clinically acceptable thresholds. Its Turing test success rate (81 ± 3%) is validated by κ = 0.85 ± 0.08, achieving very good inter-rater agreement.

CUTGAN struggled with both stain specificity and diagnostic trustworthiness. Pathologists observed severe artifacts in its virtually stained patches: severe blurring (25%), over-staining (20 ± 5%), and hallucinations (45 ± 15%). The Turing test success rate of CUTGAN was mainly restricted to 43 ± 7% due to hallucinations, fair inter-rater agreement (κ = 0.58 ± 0.15). DCLGAN preserved tissue architecture (70 ± 5%), but struggled with nuclear atypia (50 ± 15%), mitotic figure (30 ± 15%), melanin differentiation (3.9 ± 0.4), and over-staining (20 ± 5%). Cytoplasmic blurring in 40% of the cases, limited the Turing test success rate to 57 ± 6%, validated by κ = 0.68 ± 0.14, reflecting moderate inter-rater agreement.

ViT-Stain obtained the highest H&E consistency, 4.6 ± 0.1, and melanin differentiation, 4.4 ± 0.3, which is critical for melanoma diagnosis. Its diagnostic trustworthiness remained high for nuclear atypia, 80 ± 5%, tissue architecture, 90 ± 3%, and mitotic figure accuracy at 75 ± 7%. ViT-Stain patches showed mild blurring and hallucinations (10%). These factors summed up to a higher Turing test success rate (85 ± 2%), further validated by κ = 0.88 ± 0.08, indicating a near perfect inter-rater agreement. ViT-Stain consistently received the highest grades from dermatopathologists on stain specificity, diagnostic trustworthiness, and Turing test success, compared to GANs. ViT-Stain’s virtual patches and WSIs, remained comparatively realistic and similar to H&E images in fidelity. ViT-Stain’s inter-rater agreement and Turing test success rates (0.88 ± 0.08, 85 ± 2%) also closely matched those achieved by real H&E images (0.94 ± 0.05, 92 ± 2%).

### Ablation experiment

We measured the impact of each part of the ViT-Stain architecture by running an ablation experiment on the test set, keeping all hyperparameters the same (512 × 512 input, 16 × 16 patches, 12 layers, 12 heads, CNN decoder, composite loss). We tested five model variations, as shown in Table 6, and evaluated them using SSIM, PSNR, FID, and HSFI. The results show that positional embeddings are important, leading to about a 2.1% increase in SSIM and a 6% increase in PSNR. Using a hybrid CNN decoder improves PSNR by about 5% and FID by about 52% compared to using only a linear decoder. This highlights the value of local upsampling and skip connections. Decreasing the number of heads or layers degrades performance, but ViT-Stain still remains superior to all GAN baselines, suggesting some redundancy and scope for model compression. Larger tokens (32 × 32) cause a slight decrease in performance to capture finer details at the cellular level features.

**Table 6:** Results of ablation experiment evaluated on five model variants versus full ViT-Stain.

Model Variant	PE	HD	Heads	Layers	Tokens	SSIM	PSNR	FID	HSFI
**Full ViT-Stain**	**✓**	**✓**	**12**	**12**	**16** ** × 16**	**0.96**	**29.93**	**21.2**	**0.91**
PE	✗	✓	12	12	16 × 16	0.94	28.10	35.2	0.85
HD	✓	✗	12	12	16 × 16	0.94	28.50	32.3	0.86
AH (6)	✓	✓	6	12	16 × 16	0.95	29.00	27.1	0.88
Layers (8)	✓	✓	12	8	16 × 16	0.95	29.20	25.4	0.89
32 × 32 Tokens	✓	✓	12	12	32 × 32	0.95	29.40	23.3	0.90

•PE are positional embeddings.

•HD is hybrid CNN decoder.

•AH are attention heads.

### Diagnostic potential of ViT-Stain

**Fig 9:**
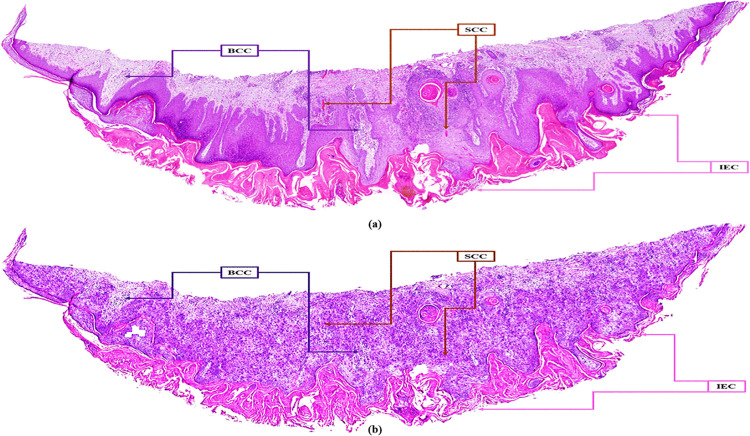
Both images represent the lesions used in the classification experiment to demonstrate the diagnostic potential of ViT-Stain. The top image **(a)** presents those lesions on H&E images, while the bottom image **(b)** displays the same lesion classes on ViT-Stain generated images; training patches were extracted from both to train the respective classifiers.

**Fig 10:**
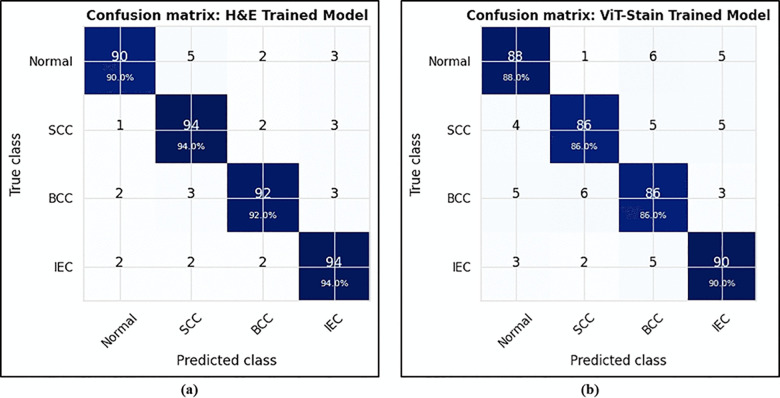
Confusion matrices of each classifier showing per-class and overall classification accuracy on the held-out test set. More precisely, the image on the left **(a)** represents the confusion matrix for the H&E classifier, and the right one **(b)** shows the confusion matrix for the ViT-Stain classifier.

**Fig 11:**
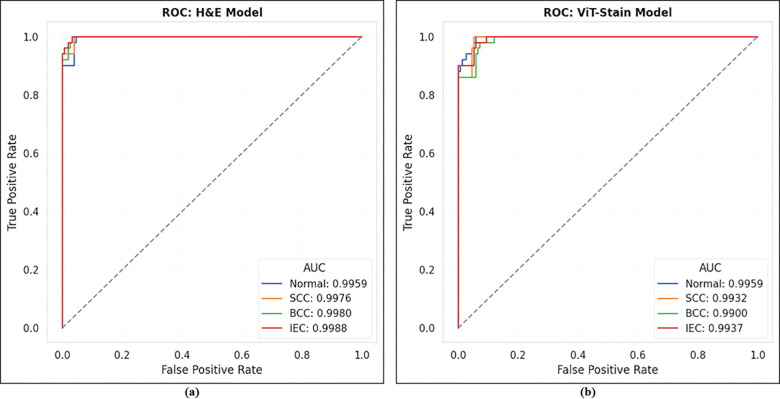
The image shows the ROC curves for the respective classifiers on the held-out test set, highlighting the true positive rate, false positive rate, and AUC. The left image **(a)** highlights the ROC curve for the H&E classifier, while the right image **(b)** shows the ROC curve for the ViT-Stain classifier.

**Fig 12:**
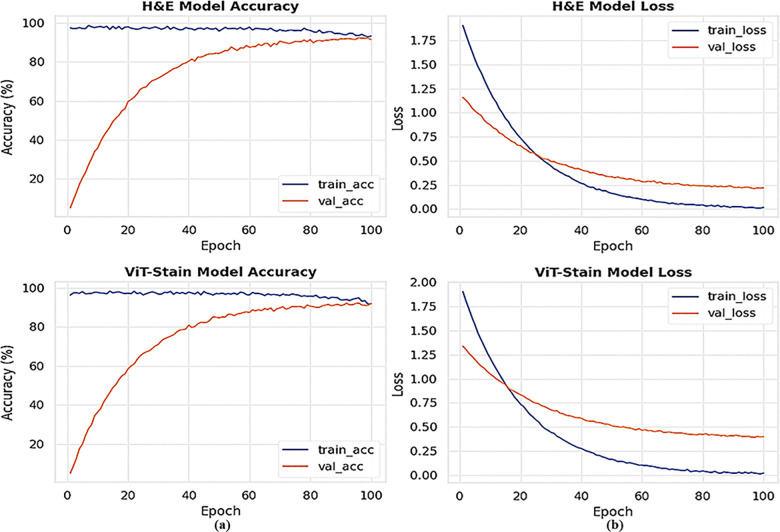
Training and validation curves for accuracy and loss for each classifier on the held-out test set are presented. The top panel **(a)** presents the curves for the H&E classifier, while the bottom panel **(b)** displays the curves for the ViT-Stain classifier.

To demonstrate the diagnostic potential of ViT-Stain beyond just perceptual quality and to validate its role in a diagnostic workflow, we carried out a classification experiment involving four types of lesions using the same E-Staining DermaRepo dataset as shown in Fig 9. The lesions we examined were normal, SCC, BCC, and IEC. We used 500 training patches for each lesion class and 100 test patches for each class. We trained two ResNet-18 classifiers. The first classifier was trained on real H&E training patches, while the other was trained on ViT-Stain-generated training patches. However, both classifiers were tested on the same held-out test set (H&E + ViT-Stain), which included 400 patches (100 for each class), to assess their ability to generalize to virtual stains. The confusion matrix (Fig 10a) showed that the classifier trained on real H&E patches achieved per-class accuracies of 90.0%, 94.0%, 92.0%, and 94.0% for normal, SCC, BCC, and IEC, respectively, leading to an overall accuracy of 92.5% on the held-out test set. The classifier trained on ViT-Stain generated patches achieved per-class accuracies of 88.0%, 86.0%, 86.0%, and 90.0% for normal, SCC, BCC, and IEC, respectively, resulting in an overall accuracy of 87.5% on the same held-out test set, as shown in Fig 10b. The one-vs-rest ROC curves (Fig 11) display characteristic classifier performance and convergence at these levels, showing a mean area under the curve (AUC) of ∼0.99. The ROC plots indicate that most lesions cluster around similar lesion types, with a slight decrease in performance on virtual stains. Fig 12 illustrates the accuracy and loss in training versus validation for H&E and ViT-Stain models. Their performance is further evaluated based on precision, recall, and F1-score as defined by the testing phase equations in Table 7. From Table 7, it is observed that the overall performance of the H&E model remains better in classifying a skin lesion into either normal (class 1), SCC (class 2), BCC (class 3), or IEC (class 4). However, ViT-Stain obtained a slightly higher precision in SCC (class 2) due to fewer false positive predictions.

**Table 7:** The classification results of the H&E and ViT-Stain trained classifiers, emphasizing precision, recall, and F-1 score to enable a direct comparison.

Model	Precision (P)				Recall (R)				F1-score			
	$\frac{TP}{TP+FP}$				$\frac{TP}{TP+FN}$				$\text{2}*\frac{P*R}{P+R}$			
**N**	**S**	**B**	**I**	**N**	**S**	**B**	**I**	**N**	**S**	**B**	**I**
**H&E**	0.95	0.90	0.94	0.92	0.90	0.94	0.92	0.94	0.92	0.92	0.93	0.93
**ViT-Stain**	0.88	0.91	0.84	0.87	0.88	0.86	0.86	0.90	0.88	0.88	0.85	0.89

•N, S, C, and I refer to lesion patches in the normal, SCC, BCC, and IEC classes, which are the categories used by the classification model.

•TP (true positive): Patches correctly identified as their true class.

•TN (true negative): Patches correctly identified as not belonging to their true class.

•FP (false positive): Patches incorrectly identified as belonging to the true class.

•FN (false negative): Patches incorrectly identified as not belonging to the true class.

### Comparative benchmarking and evaluation

To demonstrate the effect of global self-attention compared to convolutional inductive biases, we compared ViT-Stain to a selection of contemporary image-to-image translation and stain-normalization models [47–50]. The models included supervised high-resolution c-GAN (Pix2PixHD), domain-specific stain transfer (StainGAN), the fast stain-normalization network (StainNet), and a structural-constrained pathology-aware transformer GAN (SCPAT-GAN) for direct comparison with attention-enabled features. The selection criteria considered one paired c-GAN, one unpaired domain-specific, one paired lightweight network, and one transformer hybrid. These models use either convolutional or attention-based methods. To maintain a fair comparison, we trained all models on the same data splits and employed the same procedures for tile extraction, pre-processing, and training. We tested both paired training (with matched unstained and H&E tiles) and unpaired training (using images from each domain separately). Each model was evaluated using the same quantitative metrics (SSIM, PSNR, FID, KID, LPIPS, and HSFI) and also assessed for training and inference costs. Table 8 shows a side-by-side summary of how each model performed in terms of perceptual quality, distribution similarity, diagnostic value, and computational efficiency. ViT-Stain, consistently achieved the best results. SCPAT-GAN was the next best option, with an SSIM (0.95 ± 0.005), PSNR (29.74 ± 0.23), and an LPIPS (0.060 ± 0.002). However, it required more time and resources to train (~110 hours) and to run inference (~3 minutes). StainNet was the fastest (~0.3 minutes per inference) but had lower perceptual accuracy (LPIPS: 0.44 ± 0.005) and distributional similarity (FID: 284.77 ± 7.1).

**Table 8:** Comparative outcomes of H&E stains in comparison to virtual stains from ViT-Stain and leading baseline models.

Model	SSIM	PSNR	FID	KID	LPIPS	HSFI	Cost	
							TG	IN
Pix2PixHD	0.41 ± 0.015	21.02 ± 0.22	170.87 ± 6.2	0.017 ± 0.004	0.25 ± 0.002	0.71 ± 0.02	51.67	2.22
StainGAN	0.71 ± 0.010	23.60 ± 0.17	187.95 ± 4.3	0.020 ± 0.002	0.28 ± 0.002	0.62 ± 0.01	37.33	1.42
StainNet	0.80 ± 0.008	26.60 ± 0.12	284.77 ± 7.1	0.036 ± 0.003	0.44 ± 0.005	0.70 ± 0.01	14.81	0.27
SCPAT-GAN	0.95 ± 0.005	29.74 ± 0.23	23.32 ± 0.52	0.009 ± 0.001	0.060 ± 0.002	0.85 ± 0.02	110.4	2.97
**ViT-Stain**	**0.96** ** ±** ** 0.003**	**29.93** ** ±** ** 0.27**	**21.2** ** ±** ** 0.49**	**0.007** ** ±** ** 0.001**	**0.052** ** ±** ** 0.001**	**0.91** ** ±** ** 0.02**	**93.33**	**2.90**

•TG represents the training time in hours.

•IN denote the inference latency in minutes.

## Discussion

### Quantitative evaluations

ViT-Stain clearly outperformed all GANs in quantitative evaluations in our experiment, achieving the optimal mean SSIM, PSNR & HSFI, and the minimum mean LPIPS, FID & KID. These quantitative gains of virtual histology images indicate that they are almost identical to the reference H&E images, signifying strong morphologic fidelity. Conversely, each GAN exhibited explicit trade-offs. Pix2Pix (paired c-GAN) achieved only modest SSIM (0.34 ± 0.012) when perfect alignment could not be guaranteed, reflecting its dependence on pixel-wise supervision. Pix2Pix is seen to preferentially preserve fine details to unpaired methods [51], but for this task, its performance remained far below that of ViT-Stain. CycleGAN avoided paired data and resulted in realistic addition of fine details (SSIM: 0.93 ± 0.005). CUTGAN relaxed training constraints, but at the expense of severe blurring. Comparison studies previously established that CUTGAN-synthesized virtual stains (SSIM: 0.22 ± 0.013) were blurrier, bleached, and less homogeneous than those synthesized with other GANs [7]. DCLGAN synthesized virtual stains that were less clear and detailed than those synthesized with CUTGAN, indicating reduced output fidelity.

Compared to other methods, ViT-Stain performed better by achieving lower FID, KID, and LPIPS scores. This means its results are more similar to real H&E images, supporting both perceptual and distributional realism. FID showed a strong negative correlation (r = −0.79) with pathologist scores, which indicates that lower FID values are linked to higher Turing test scores and greater perceptual realism, as shown in Table 9. While FID, KID, and LPIPS are useful for measuring realism, they cannot fully replace thorough pathologist reviews and clinical testing. Examining the limits of agreement, particularly concerning inter-rater variability as discussed by Li et al. [52], provides a better benchmark than merely using pixel-level accuracy. Ultimately, progress in virtual staining will rely on setting clear and enforceable standards for diagnostic agreement.

**Table 9:** Pearson correlation coefficient (r) between two sets of measurements: HSFI and Turing test success and FID and Turing test success for the corresponding image measurements derived from the virtual staining and real H&E frameworks.

Model	HSFI	FID	Turing Test Success (%)	Pearson Correlation Coefficient (r)	
				HSFI	FID
**Pix2Pix**	0.67 ± 0.02	256.3 ± 5.96	72 ± 4	0.92	− 0.79
**CycleGAN**	0.81 ± 0.02	27.7 ± 0.64	81 ± 3		
**CUTGAN**	0.58 ± 0.01	233.6 ± 5.43	43 ± 7		
**DCLGAN**	0.59 ± 0.01	228.3 ± 5.31	57 ± 6		
**ViT-Stain**	0.91 ± 0.02	21.2 ± 0.49	85 ± 2		
**H&E**	1	0	92 ± 2		

Medical stain translation prioritizes structural accuracy over diversity, thereby favoring the cyclic mappings offered by CycleGAN and the global contextualization presented by ViT-Stain. Both CycleGAN and ViT-Stain control hallucinations, a critical requirement for histopathology, due to cyclic constraints and MHSA, respectively [53]. In comparison, artifacts infused by CUTGAN and DCLGAN compromise HSFI below meaningful thresholds (HSFI < 0.6). In particular, the HSFI of the ViT-Stain HSFI (0.91 ± 0.02), validated with pathologist concordance, sets it up firmly as an AI contender for the grading of melanoma [54]. Furthermore, the HSFI also correlates highly with pathologist scores (r = 0.92), verifying a strong positive correlation between fidelity and diagnostic scores, as tabulated in Table 9. Here, it is seen that HSFI is aligned with human perceptual success rates, verifying clinical interpretability and consolidating it as a perceptually valid quality index for virtual staining.

ViT-Stain takes significantly longer to train (~93 hours) than GANs (~33–48 hours) for the following three main factors [33,55–57]: self-attention, scaling quadratically with the number of tokens; an overlapping patch scheme, resulting in increased floating-point operations (FLOPs) compared with non-overlapping approaches; and a very large parameter size (~86 million), increasing gradient-computation overhead at mixed-precision backpropagation compared to standard U-Net-based generators (~50 million parameters). Nevertheless, since training is an offline process, this is an acceptable trade-off for ViTs owing to superior global structure modeling and fewer artifacts. Inference time per image becomes paramount following deployment, particularly for healthcare. When models are trained and frozen, gradient computation, loss backpropagation, and weight updates are not performed anymore. Their efficacy depends on the efficiency of the forward-pass and low-latency outputs, critical for digital pathology. Pathologists demand immediate diagnostic feedback, and reducing latency enables hypothesis testing. Processing each image in 2–3 minutes slows inference and raises screening costs. Therefore, although performance improvement matters, but efficient inference is just as crucial for clinical use.

Hence, we need to consider clinical benefits of ViT-Stain beyond training time. Assess its real-world suitability: workflow integration, pathologist usability, and patient result turnaround, all of which depend on inference speed. Slow inference reduces the value of pathologists’ work. For fast and accurate diagnosis, inference should take only a few seconds per slide, as shown in [58–59]. We note ViT-Stain’s high processing demands may hinder its deployment in resource-limited settings. To enhance the feasibility of ViT-Stain in such environments, we plan to adopt a prioritized strategy in the future to reduce computational demands. This strategy will include knowledge distillation to compress large models with minimal loss in accuracy [60–62], structured pruning and sparsity-aware training to reduce FLOPs and enhance execution speed on standard inference platforms [63], quantization-aware training (QAT) to preserve fidelity [64], and progressive resizing that starts training at a smaller patch size of 256 × 256 and progressively increases to 512 × 512 in the final training epoch. The ablation experiment also confirms that each tailored modification, plays a critical role in achieving ViT-Stain’s high-fidelity virtual staining. Moreover, it also identifies promising avenues for efficiency optimizations with minimal performance loss.

The results from the classification experiment show that ViT-Stain images retain most of the class-discriminative signal, especially precision in identifying SCC (class 2). They can be used in downstream classification processes, with only a small decrease in performance. The accuracy of both the classifiers trained with H&E staining and ViT-Stain is almost similar, differing by only 5%. These findings highlight two key points. First, ViT-Stain’s virtual stains provide local texture and color changes that modern convolutional classifiers use to identify lesions, supporting timely and reliable diagnostic workflows. Second, the minor performance drops are mostly noticeable in clinically important cases, particularly between closely related classes. Our model aligns with existing research in the field of computational pathology, including the usage of deep features, stringent external validation, and assessing the translation of images. Nevertheless, DL classifiers must be evaluated under the same image conditions that are anticipated at deployment time [19,65-66]. Our classification results also correlate with inter-rater agreement and visual Turing test assessments, performed by expert dermapathologists comparing H&E and ViT-Stain images.

ViT-Stain’s global attention mechanism achieves a significant improvement in SSIM, FID, and LPIPS scores. This is validated through comparison with the leading baselines. Virtual staining benefits from exposure to long-range tissue context and color assignments over large spatial areas. Conversely, the pix2pixHD model lacks global content-adaptability provided by attention mechanisms, since it is convolutional. Its FID (170.87 ± 6.2) is higher due to poor global structural consistency with respect to transformer-based models. The training (∼51.67 hours) and inference times (∼2.22 minutes) are higher compared to Pix2Pix (∼33.33 hours, ∼1.6 minutes) due to the multi-scale operations. StainGAN reduces major color artifacts and improves color realism. It is easier to train (∼37.33 hours) and is lighter at inference (∼1.42 minutes). Nonetheless, its FID (187.95 ± 4.3) and LPIPS (0.28 ± 0.002) scores are marginally less advantageous compared to those of Pix2PixHD (170.87 ± 6.2, 0.25 ± 0.002) when structural context is required over long distances. StainNet is designed for speed and is suitable for deterministic stain normalization, trading some perceptual accuracy for efficiency (FID: 284.77, LPIPS: 0.44). Its training takes 14.81 hours, while inference takes 0.27 minutes, making it ideal for resource-limited scenarios despite not having the highest accuracy. SCPAT-GAN, by contrast, is a hybrid model utilizing transformer-based global attention, convolutional decoding, and adversarial training. It offers almost similar perception and structural gains as ViT-Stain but at a higher computational cost (training: 110.40 hours; inference: 2.97 minutes), fitting scenarios with greater resources and tolerance for complexity. The potential of transformer-based generators has also been noted in other fields. For example, a Swin Transformer-GAN has achieved higher fidelity in multimodal medical imaging compared to the standard Pix2Pix and CycleGAN. Consequently, a ViT-GAN produced more realistic translated images than conventional CNNs [67–68].

Overall, ViT-Stain’s strong performance stems from its architectural design. The transformer backbone enables global MHSA across the entire image, allowing the model to simultaneously capture long-range tissue context and fine-grained details. Nevertheless, attention alone is not sufficient for producing high-fidelity stain synthesis. Local fine-grained details, such as fine chromatin texture and nuclear edges, are also significant. Our hybrid design integrates a global transformer encoder with a convolutional decoder. The transformer encoder enhances virtual staining by enabling content-adaptive context and global aggregation of histological details. Consequently, the model resolves local ambiguities and preserves tissue-level coherence, a capability that CNNs are only able to achieve indirectly through deep layers or expensive architectural schemes. Our convolutional decoder recovers fine spatial details with the help of local convolutional upsampling and texture cues. On the other hand, convolutional GANs are inherently local in nature. Recent literature has also indicated that CNN-based translation networks struggle to preserve global features and capture long-range dependencies efficiently [27].

### Qualitative evaluations

ViT-Stain exhibits a higher Turing test success rate (~85%) than Pix2Pix, CUTGAN, and DCLGAN, confirming its clinical potential. It produces virtual stains with fewer artifacts and better melanin differentiation, as supported by recent studies [69] showing the effectiveness of the global MHSA mechanism in capturing subtle color and shape details in melanocytic lesions. For instance, CUTGAN tends to create more artifacts, which lowers Fleiss’ κ, increases inconsistency, and diagnostic risk in pathology. DCLGAN preserves structural details but struggles to accurately distinguish melanin [70], making it less suitable for analyzing pigment-sensitive areas.

ViT-Stain defines cell boundaries more clearly, shows more consistent H&E staining, and reduces the typical image hallucinations seen with GANs. In qualitative assessments, pathologists preferred images from ViT-Stain, noting better diagnostic accuracy, thanks to improved morphological details and fewer false-positive staining artifacts. These strengths help ViT-Stain address key issues with CNNs, like inaccurate boundaries and color distortions. This suggests ViT-Stain can improve interpretability and build clinicians trust in virtual staining. Performance reviews also show that ViT-Stain can complement current DL models in digital pathology for better precision, clarity, and consistency [71]. Recent studies also support ViT-Stain’s role in improving image and diagnostic quality in virtual staining [72–73].

Overall, ViT-Stain should be considered the preferred approach for digital histopathology when accuracy is paramount, as it offers new dimensions by integrating a ViT encoder with a convolutional decoder. The HSFI measures diagnostic accuracy, matching expert assessments, and making comparisons more reliable. ViT-Stain’s performance in classification tasks, supports its potential in real diagnostic settings, even when perceptual quality varies. Our careful training, including data preparation, parameter tuning, registration, and patching, supports its strong results. However, ViT-Stain requires more computational resources and can lead to overfitting if training data is limited. ViT-Stain’s strong diagnostic accuracy but with higher resource demands, makes it most valuable where accuracy is the primary concern.

## Limitations & future work

ViT-Stain has shown promising results with various tissue samples, but it does have some limitations. The model was trained and tested on pairs of unstained and H&E-stained images from the E-Staining DermaRepo, which primarily focuses on skin tissue. Therefore, to ensure reliable performance with other staining protocols, such as IHC or stains like PAS and Masson’s trichrome, it may require either retraining or fine-tuning the model. Variations due to digitization hardware, slide scanning parameters, or laboratory protocols may give rise to invariant color shifts in texture across different centers. Exposure to previously untested scanner models or varying staining protocols might compromise its performance [74–76]. The color deconvolution and stain transfer procedure can be misled by the overlapping spectra from high pigment content, e.g., melanin and hemosiderin, potentially causing degradation for segmentation or NMS. In the course of our assessments, we also observed some failure instances among melanin-dominant lesions. The quadratic scaling inherent to self-attention causes the model to be computationally expensive, particularly at processing high-resolution patch locations derived from gigapixel WSIs [77–78]. The patch and merge process can decrease inference speed and prevent real-time processing for clinical scanners.

To stringently test domain shift, we plan to perform external multi-center validation with cohorts that are digitized using diverse scanner models and staining laboratories [19,65]. We will address domain variability through the use of adaptive instance distribution alignment (AIDA), stain-specific normalization, and CycleGAN-based unpaired domain translation to match cross-center distribution [79]. Transforming ViT-Stain into a multi-domain transformer conditioned on stain type or by using multi-task learning for variable appearances may improve generalization. Efficient transformers, including Linformer or Performer, and hierarchical window-based architecture, such as Swin-Transformer [27,80,81], and variants of ViT, including MobileViT or DeiT, may minimize computational overhead with retention of context modeling [29,82]. Finally, the deployment of ViT-Stain within a WSI viewer also necessitates compliance with regulatory requirements, including the TRIPOD-AI checklist.

## Conclusion

ViT-Stain pushes virtual histological staining by integrating transformer-based global context modeling and high-resolution convolutional decoding. This approach attains robust performance levels in terms of structure, perception, and diagnosis. Through the adoption of MHSA, ViT-Stain achieves high-fidelity reproduction of tissue morphology, texture, color homogeneity, and cellular attributes, including nuclear granulation, verified with expert dermatopathologists. The novel HSFI significantly correlates with expert scores, structural, and perceptual realism. Together, these results ascertain the potential of ViTs to address receptive field limitations of earlier CNNs/GANs, and point to the deployment of context-aware AI for computational pathology.

Despite not being a turnkey solution to the substitution of chemical staining for all tissue classes or platforms, ViT-Stain represents a significant technical step toward practical virtual staining for research and certain clinical workflows. The suitability of transformer-based virtual staining for diagnostic evaluation, justify further clinical validation. In future work, we will build on these findings to work with additional stain types, integrate multi-site datasets, and add domain adaptation for improved robustness. Addressing the pervasive ethical and technical issues relating to the deployment of AI continues to be critical to increase accessibility to precision medicine.
